# Listening to the voices of the medically silenced

**DOI:** 10.1371/journal.pgph.0003761

**Published:** 2024-10-08

**Authors:** Rageshri Dhairyawan

**Affiliations:** Department of Infection and Immunity, Barts Health NHS Trust, London, United Kingdom; PLOS: Public Library of Science, UNITED STATES OF AMERICA

## Introduction

*“There’s really no such thing as the ‘voiceless*.*’ There are only the deliberately silenced*, *or the preferably unheard”* said Arundhati Roy on accepting the Sydney Peace Prize in 2004 [1]. When it comes to global health, whose voices are we not hearing? A recent study by Nassiri-Ansari and colleagues gives us a clue [2]. They analysed 1269 articles published in twelve medicine, global health and infectious diseases journals between 2019–2021, by authorship status. They found that the majority of the prestigious first and last author positions were occupied by men from institutions affiliated with high-income countries (HIC). This was even when the papers focused on low- and middle-income countries (LMIC). This points to a silencing of researchers from LMIC which does not come as a surprise. The dominance of academics from high income countries in the Global North is well-documented [3]. They are more likely to lead global health institutions, set the research agenda, be awarded funding, be published, cited and participate in international conferences. Scientists from the Global South find their credibility as knowers questioned [4]. This results in research that may not be meaningful for communities in the Global South, reinforcing knowledge gaps and limiting our collective evidence base. This has rightly led to calls to challenge the historical legacies and ongoing practices of colonialism in global health [5].

In a recent book, I discuss how the medical practice of silencing is a systemic issue that extends further than global health to every level of healthcare and research [3]. It predominantly affects the same minoritised communities that experience health inequities as well as other forms of social injustice, and exacerbates them.

## Systemic silencing

This silencing starts in the consultation room and on hospital wards. In patient safety investigations, a common theme is that patients and their carers report not being listened to, taken seriously or believed when they report their concerns to healthcare professionals. This is seen most starkly in maternal mortality reports where in the UK for example, Black women are 4x and Asian women 2x more likely to die than white women [6]. They are regarded as untrustworthy and more likely to be doubted due to their ethnicity and gender. They experience epistemic injustice, a wrong occurring to them in their capacity as knowers due to stereotypes about their identity [7]. This can also be due to social class, sexual orientation, ability, age and religion. While minoritised patients are least likely to be listened to, to an extent all patients with long-term health conditions may be seen as unreliable narrators, *because* they are ill. They may be stereotyped as being incapable, incompetent and unable to be objective about their illness [8]. When healthcare professionals disbelieve and dismiss patients, this can have devastating impacts including delayed diagnosis, treatment and even death. Repeated incidents of not being heard silences patients, deterring them from speaking up for fear of further rejection, fuelling mistrust and healthcare avoidance [3,9].

Doctors learn to be sceptical of patients in their training. This is even ingrained in medical language with phrases such as “she claimed”, “she denied” commonplace [10]. Patients with conditions for which there are few objective signs, diagnostic criteria and known effective treatments such as fibromyalgia and myalgic encephalomyelitis/chronic fatigue syndrome (ME/CFS) often report they are dismissed or shut down by doctors [11]. Diagnosis relies on patient testimony rather than laboratory or radiological investigations, which is viewed as inherently untrustworthy. Doctors prefer certainty–diseases where the pathophysiology is understood, diagnosis straightforward and potentially curable, such as myocardial infarctions [12]. Consequently, patients with conditions such as fibromyalgia experience epistemic injustice and become further marginalised as these receive less research and policy attention, leaving knowledge gaps unfilled.

When patients are silenced, healthcare professionals can be powerful advocates. However, the medical practice of silencing extends to the workforce with minoritised healthcare professionals often reporting a culture of exclusion in their institutions, suffering prejudice, discrimination and doubts about their credibility [13]. Repeatedly going unheard can impact on self-esteem and career progression, making it is less likely that they apply for leadership positions with the authority to decide on funding and policy [14]. This results in the minoritised communities they advocate for continuing to be under-served. We see a similar picture with minoritised researchers in the Global North who are less likely to be awarded grants, be published and be promoted to professorship, affecting who and what is researched, often to the detriment of marginalised groups [3].

## A healthcare system that listens

A common solution given to being silenced is to speak up louder. But this is not always possible especially for patients who are at their most vulnerable when unwell, or who may be deemed ‘aggressive’ due to social stereotypes. Instead, we in healthcare and research need to learn to listen better and do so in ways that do not cause further harm. This includes acknowledging the inherent power imbalance between doctors and patients and more highly valuing patients’ expertise in their own bodies. As well as addressing individual and institutional bias, listening needs to be prioritised in medical education and in healthcare services, recognising its value as a healing tool in itself for patients. Doctors need to understand that they may not be able to ‘fix’ all of their patients, but bearing witness to their suffering in itself, may be therapeutic.

Patient voice has been downgraded in medicine and research for centuries [15]. We see this in the hierarchy of evidence, which excludes qualitative research and other methods foregrounding patient voice. However, patients have fought back collectively to be heard and created real change, a prominent example being HIV/AIDS activists. If we are serious about improving healthcare, we must include patients at every level in education, policy, service development and research. They should be offered adequate renumeration, training, supervision and pastoral support, and shown their input has resulted in action.

To address health inequity, it is increasingly urgent that we listen to and amplify the voices of those who have been historically silenced. This will require a shift in power from those who have benefitted from their silence. Are we ready to listen?
